# Prognostic significance of XRCC4 expression in hepatocellular carcinoma

**DOI:** 10.18632/oncotarget.21360

**Published:** 2017-09-28

**Authors:** Jun Lu, Xing-Zhizi Wang, Tian-Qi Zhang, Xiao-Ying Huang, Jin-Guang Yao, Chao Wang, Zhong-Hong Wei, Yun Ma, Xue-Min Wu, Chun-Ying Luo, Qiang Xia, Xi-Dai Long

**Affiliations:** ^1^ Department of Liver Surgery, Ren Ji Hospital, School of Medicine, Shanghai Jiao Tong University, Shanghai 200127, P.R.China; ^2^ Department of Pathology, Youjiang Medical University for Nationalities, Baise 533000, P.R.China; ^3^ Department of Medicine, The Affiliated Hospital of Youjiang Medical University for Nationalities, Baise 533000, P.R.China; ^4^ Department of Tumor, The Affiliated Hospital of Youjiang Medical University for Nationalities, Baise 533000, P.R.China; ^5^ Department of Pathology, The Affiliated Tumor Hospital of Guangxi Medical University, Nanning 530021, P.R.China

**Keywords:** XRCC4, hepatocarcinoma, prognosis, TACE

## Abstract

**Background:**

Our previous investigations have shown that the variants of X-ray repair complementing 4 (XRCC4) may be involved in hepatocellular carcinoma (hepatocarcinoma) tumorigenesis. This study aimed to investigate the possible prognostic significance of XRCC4 expression for hepatocarcinoma patients and possible value for the selection of transarterial chemoembolization (TACE) treatment.

**Materials and Methods:**

We conducted a hospital-based retrospective analysis (including 421 hepatocarcinoma cases) to analyze the effects of XRCC4 on hepatocarcinoma prognosis and TACE. The levels of XRCC4 expression were tested using immunohistochemistry. The sensitivity of cancer cells to anti-cancer drug doxorubicin was evaluated using the half-maximal inhibitory concentration (IC50).

**Results:**

XRCC4 expression was significantly correlated with pathological features including tumor stage, liver cirrhosis, and micro-vessel density. XRCC4 expression was an independent prognostic factor of hepatocarcinoma, and TACE treatments had no effects on prognosis of hepatocarcinoma patients with high XRCC4 expression. More intriguingly, TACE improved the prognosis of hepatocarcinoma patients with low XRCC4 expression. Functionally, XRCC4 overexpression increased while XRCC4 knockdown reduced the IC50 of cancer cells to doxorubicin.

**Conclusions:**

These results suggest that XRCC4 may be an independent prognostic factor for hepatocarcinoma patients, and that decreasing XRCC4 expression may be beneficial for post-operative adjuvant TACE treatment in hepatocarcinoma.

## INTRODUCTION

Hepatocarcinomas, also called liver cell carcinoma or hepatocellular carcinoma, is very common in the People's Republic of China, and the incidence and mortality of this cancer has increased significantly in past decades [1, 2]. Although the advances in surgical techniques, including hepatectomy, liver transplantation, and perioperative management, make it an important candidate as curative therapy method for hepatocarcinoma, long-term survival is still unsatisfactory due to the high tumor recurrence rate after curative treatment [1–3]. Recent clinic studies have shown that transarterial chemoembolization (TACE) enjoys the advantages of increasing local anti-cancer drug concentrations and reducing systemic adverse reaction. This treatment is determined as an attractive conservative therapy for hepatocarcinoma cases, especially these with advanced-stage hepatocarcinoma [4–8]. However, increasing evidence has proved that hepatocarcinoma patients having different genetic profiles will exhibit different therapeutic response to this therapy [8–13]. Therefore, it is crucial for patients with hepatocarcinoma to need a prognostic marker identifying whether they are at high recurrence risk or can display therapeutic response to TACE.

XRCC4, an important non-homologous end-joining repair gene, can interact directly with Ku70/Ku80 complex and serve as a crucial scaffold protein between this complex and DNA Ligase IV in the process of DNA double-strand break repair pathway [14, 15]. Recently, some studies have reported that the loss of XRCC4 function involves in carcinogenesis of some cancers, and can modify the sensitivity of cancer cells to chemotherapy and radiotherapy [16–19]. Our previous reports have also shown that the genetic variable of this gene is correlated with increasing risk and poor survival of hepatocarcinoma [20, 21]. Here, we continue to study association between XRCC4 expression in cancerous tissues and the survival of hepatocarcinoma patients, and to explore potential significance in selecting TACE treatment.

## RESULTS

### The clinicopathological features of cases with hepatocarcinoma

The clinicopathological features of the patients are shown in Table 1. A total of 421 patients with hepatocarcinoma were included in the present study with an average age of 47.8 ± 10.2 years. The HBV and HCV infective rates were 73.9% (311 of 421) and 11.6% (49 of 421), respectively. About 40% of hepatocarcinoma cases were in the 0 –A stage of the Barcelona Clinic Liver Cancer (BCLC) staging system, and accepted the radical surgical therapy. At a median follow-up of 60 months, 257 featured tumor recurrence with 30.00 (22.11-37.89) months of median tumor recurrence-free survival time (MRT), and 266 died with 45.00 (38.88-51.13) months of median overall survival time (MST).

**Table 1:** The association between XRCC4 expression and clinic-pathological features of hepatocarcinoma

Variables	Cases, n (%)	XRCC4 expression, n (%)		OR (95% CI)	*P*_trend_
		High	Low		
Total	421 (100%)	241 (100%)	180 (100%)		
Age (yrs)					
≤ 48	233 (55.3)	133 (55.2)	100 (55.6)	Reference	
> 48	188 (44.7)	108 (44.8)	80 (45.4)	1.01 (0.68-1.49)	0.97
Sex					
Man	285 (67.7)	161 (66.8)	124 (68.9)	Reference	
Female	136 (32.3)	80 (33.2)	56 (31.1)	1.10 (0.73-1.67)	0.66
Ethnicity					
Han	225 (53.4)	124 (51.5)	101 (56.1)	Reference	
Zhuang	196 (46.6)	117 (48.5)	79 (43.9)	1.16 (0.75-2.69)	0.51
HBsAg					
Negative	110 (26.1)	65 (27.0)	45 (25.0)	Reference	
Positive	311 (73.9)	176 (73.0)	135 (75.0)	0.91 (0.55-1.50)	0.69
anti-HCV					
Negative	372 (88.4)	214 (88.8)	158 (87.8)	Reference	
Positive	49 (11.6)	27 (11.2)	22 (12.2)	0.75 (0.37-1.50)	0.41
Smoking status					
No	309 (73.4)	179 (74.3)	130 (72.2)	Reference	
Yes	112 (26.6)	62 (25.7)	50 (29.8)	0.90 (0.30-2.69)	0.85
Drinking status					
No	298 (70.8)	175 (72.6)	123 (68.3)	Reference	
Yes	123 (29.2)	66 (27.4)	57 (31.7)	0.90 (0.31-2.61)	0.84
AFP (ng/mL)					
≤ 20	154 (36.6)	83 (29.5)	71 (39.4)	Reference	
> 20	267 (63.4)	158 (70.5)	109 (60.6)	1.17 (0.75-1.84)	0.42
Liver cirrhosis					
No	103 (24.5)	74 (30.7)	29 (16.1)	Reference	
Yes	318 (75.5)	167 (69.3)	151 (83.9)	2.01 (1.18-3.42)	0.01
Tumor size					
≤3 cm	207 (49.2)	120 (49.8)	87 (48.3)	Reference	
> 3 cm	214 (50.8)	121 (50.2)	93 (51.7)	1.15 (0.74-1.29)	0.54
Tumor grade					
Low grade	223 (53.0)	132 (54.8)	91 (50.6)	Reference	
High grade	198 (47.0)	109 (45.2)	89 (49.4)	0.79 (0.51-1.43)	0.28
BCLC stage					
0-A	164 (39.0)	131 (54.4)	33 (18.3)	Reference	
B-C	257 (61.0)	110 (45.6)	147 (81.7)	4.98 (3.07-8.06)	6.71 × 10^−11^
MVD					
Negative	188 (44.7)	131 (54.4)	57 (31.7)	Reference	
Positive	233 (55.3)	110 (45.6)	123 (68.3)	2.23 (1.43-3.47)	4.03 × 10^−4^

### XRCC4 expression correlated with clinicopathological features of hepatocarcinoma

**Figure 1:**
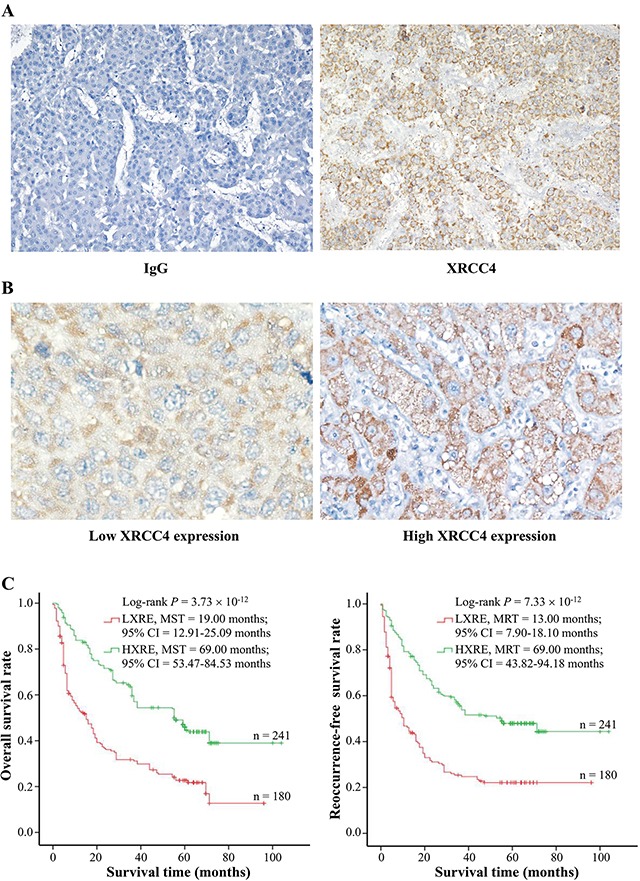
The association between XRCC4 expression and hepatocarcinoma prognosis in the 421 cases **(A)** Specificity of anti-XRCC4 antibody for immunohistochemistry (IHC) staining. IHC staining with anti-XRCC4 (Right) and nonrelated IgG (**Left**) was used to analyze the specificity of anti-XRCC4. In the tissues with hepatocarcinoma, XRCC4 protein mainly localized at cytoplasm. **(B)** The expression levels of XRCC4 protein in the cancerous tissues were elucidated using immunohistochemistry scores of IRS system (*See Materiel and methods*). According to the median expression level in cancerous tissues, the levels of XRCC4 expression were divided into two groups: low expression group (IRS ≤ 4) and high expression group (IRS > 4). Representative images show different expression levels (Original magnification × 400). **(C)** The XRCC4 expression significantly modified overall survival (Left) and tumor recurrence-free survival (Right). Cumulative hazard function was plotted by Kaplan-Meier's methodology, and *P* value was calculated with two-sided log-rank tests. *Abbreviation*. HXRE, high XRCC4 expression; LXRE, low XRCC4 expression; MST, the median overall survival time; MRT, the median tumor reoccurrence-free survival time.

To investigate the correlation between XRCC4 expression and clinicopathological characteristics of hepatocarcinoma cases, we detected the expression of XRCC4 protein in tumor tissues using immunohistochemistry technique. In this study, the specificity of anti-XRCC4 was first elucidated, and results showed this antibody can specifically recognize XRCC4 protein (Figure 1A). The immunohistochemistry staining exhibited that a minority of XRCC4 proteins were expressed in the nucleus of cancer cells, and most were localized at their cytoplasm. To analyze, the XRCC4 protein-expressing levels were separated into two groups: high XRCC4 expression (HXRE) group and low XRCC4 expression (LXRE) group, according to the median expression (Figure 1B). Results from logistic regression analyses displayed that the XRCC4 protein-expressing levels were significantly associated with liver cirrhosis [odds ratio (OR) = 2.01, 95% confidence interval (CI) = 1.18-3.42], tumor stage (OR = 4.98, 95% CI = 3.07-8.06), and microvessel density (OR = 2.23, 95% CI = 1.43-3.47), but not to other clinicopathological characteristics (Table 1).

### XRCC4 expression significantly affected the outcome of cases with hepatocarcinoma

Although our previous reports exhibited that the genic mutations at coding regions of XRCC4 were significantly related to poor overall survival (OS) and tumor recurrence-free survival (RFS) of hepatocarcinoma cases [20, 21], it is not still clear whether XRCC4 expression is an independent marker for hepatocarcinoma prognosis. To answer it, we first performed Kaplan-Meier survival analysis and found these cases with HXRE in their tumor tissues had a longer MST and MRT compared to those with low expression (69.00 vs. 19.00 months for MST; 69.00 vs. 13.00 months for MRT) (Figure 1C). Next, we accomplished a series of univariate analyses based on Cox Regression Model (with an Enter Method) and found these hepatocarcinoma patients with LXRE in their cancerous tissues featured an increasing death risk [hazard ratio (HR) = 2.26 and 95% CI = 1.77-2.89] and tumor-recurrence risk (HR = 2.21 and 95% CI = 1.72-2.83) compared to those with HXRE in the tumor tissues (Table 2). This was indicative of XRCC4 expression ameliorating survival of patients with hepatocarcinoma.

**Table 2:** Univariate analyses identify XRCC4 expression as one of significant prognostic predictors for survival of patients with hepatocarcinoma

Variables	OS		RFS	
	HR (95% CI)	*P*_trend_	HR (95% CI)	*P*_trend_
Age (48 vs. <48 yrs)	0.85 (0.67-1.09)	0.20	0.81 (0.63-1.23)	0.29
Gender (Female vs. Male)	0.89 (0.69-1.16)	0.39	0.88 (0.68-1.16)	0.38
Ethnicity (Minority vs. Han)	0.89 (0.70-1.13)	0.34	0.93 (0.73-1.19)	0.55
Smoking (Yes vs. No)	1.06 (0.81-1.39)	0.66	1.05 (0.79-1.38)	0.75
Drinking (Yes vs. No)	1.16 (0.89-1.51)	0.27	1.14 (0.87-1.49)	0.34
HBsAg (Positive vs. Negative)	1.00 (0.76-1.32)	0.99	1.00 (0.76-1.32)	0.99
anti-HCV (Positive vs. Negative)	0.84 (0.57-1.25)	0.40	0.88 (0.59-1.30)	0.50
AFP (≤ 20 vs. > 20 ng/mL)	0.91 (0.71-1.16)	0.44	0.87 (0.67-1.11)	0.26
Liver cirrhosis (Yes vs. No)	1.45 (1.08-1.94)	0.01	1.52 (1.12-2.05)	7.16 ×10^−3^
tumor size (≤ 3 vs. > 3 cm)	2.12 (1.65-2.71)	3.66×10^−9^	2.15 (1.67-2.77)	3.27×10^−9^
Tumor grade (High vs. Low)	1.05 (0.83-1.34)	0.69	1.06 (0.83-1.36)	0.63
BCLC stage (B-C vs. 0-A)	4.26 (3.19-5.69)	7.54×10^−23^	4.24 (3.15-5.72)	2.21×10^−21^
MVD (Positive vs. Negative)	1.78 (1.39-2.27)	5.00×10^−6^	1.61 (1.26-2.07)	1.83×10^−4^
XRCC4 expression (Low vs. High)	2.26 (1.77-2.89)	7.56×10^−11^	2.21 (1.72-2.83)	4.98×10^−10^

Abbreviations: CI, confidence interval; HR, hazards ratio, OS, overall survival; RFS, tumor reoccurrence-free survival; MVD, microvessel density.

To investigate whether predictive value of XRCC4 expression for hepatocarcinoma survival was regulated by known clinicopathological features (including age, gender, minority, smoking and drinking status, HBV and HCV infective status, AFP, liver cirrhosis, tumor size, tumor differentiation grade, micro-vessel amount, and BCLC stage), a multivariate survival analysis on the basis of Cox Regression Model (with an Enter Method) was finished (Table 3). Like some known predictive markers such as tumor size, tumor stage, and microvessel density [11, 22], XRCC4 expression was significantly associated with the prognosis of hepatocarcinoma, and the corresponding prognostic values were 1.63 (1.25-2.11) for OS and 1.55 (1.19-2.02) for RFS, respectively (Table 3). All together, these data indicated that XRCC4 expression in the cancerous tissues could serve as a significant predictive marker for cases with hepatocarcinoma, and that this predictive significance should not be regulated by other clinicopathological features.

**Table 3:** Independent prognostic factors of OS and RFS for patients with hepatocarcinoma by multivariate analyses

Variables	OS		RFS	
	HR (95% CI)	*P*_trend_	HR (95% CI)	*P*_trend_
Age (48 vs. <48 yrs)	0.76 (0.59-1.18)	0.12	0.71 (0.55-1.22)	0.11
Gender (Female vs. Male)	0.89 (0.68-1.16)	0.38	0.89 (0.67-1.17)	0.39
Ethnicity (Minority vs. Han)	1.00 (0.78-1.28)	0.99	1.00 (0.78-1.29)	0.99
Smoking (Yes vs. No)	0.66 (0.35-1.24)	0.20	0.69 (0.36-1.33)	0.27
Drinking (Yes vs. No)	1.64 (0.89-3.03)	0.11	1.46 (0.78-2.75)	0.24
HBsAg (Positive vs. Negative)	1.07 (0.81-1.43)	0.63	1.05 (0.79-1.40)	0.74
anti-HCV (Positive vs. Negative)	0.80 (0.53-1.20)	0.29	0.89 (0.59-1.35)	0.60
AFP (≤ 20 vs. > 20 ng/mL)	1.08 (0.84-1.40)	0.54	1.03 (0.80-1.34)	0.80
Liver cirrhosis (Yes vs. No)	1.38 (1.02-1.87)	0.04	1.39 (1.02-1.89)	0.04
tumor size (≤ 3 vs. > 3 cm)	2.27 (1.76-2.94)	4.37×10^−10^	2.18 (1.68-2.82)	4.50×10^−9^
Tumor grade (High vs. Low)	1.13 (0.88-1.45)	0.34	1.17 (0.91-1.51)	0.23
BCLC stage (B-C vs. 0-A)	3.92 (2.90-5.31)	1.06×10^−18^	3.77 (2.75-5.17)	1.71×10^−16^
MVD (Positive vs. Negative)	1.68 (1.29-2.19)	1.37×10^−4^	1.47 (1.12-1.91)	4.70×10^−3^
XRCC4 expression (Low vs. High)	1.63 (1.25-2.11)	2.47×10^−4^	1.55 (1.19-2.02)	1.07×10^−3^

Abbreviations: CI, confidence interval; HR, hazards ratio, OS, overall survival; RFS, tumor reoccurrence-free survival; MVD, microvessel density.

### XRCC4 expression differently modified hepatocarcinomas’ response to TACE treatment

**Figure 2:**
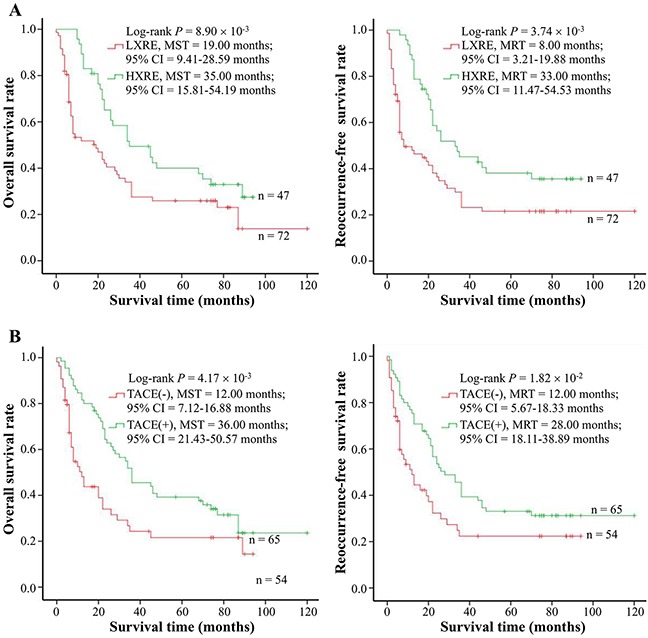
The effects of XRCC4 expression and TACE on hepatocarcinoma prognosis in 119 cases with BCLC B-stage hepatocarcinoma **(A)** The expression levels of XRCC4 protein in the cancerous tissues were associated with overall survival (Left) and tumor reoccurrence-free survival (**Right**) of hepatocarcinoma cases. **(B)** TACE treatment was related to overall survival (Left) and tumor reoccurrence-free survival (**Right**) of hepatocarcinoma cases. Cumulative hazard function was plotted by Kaplan-Meier's methodology, and *P* value was calculated with two-sided log-rank tests. *Abbreviations*. HXRE, high XRCC4 expression; LXRE, low XRCC4 expression; MST, the median overall survival time; MRT, the median tumor reoccurrence-free survival time.

**Figure 3:**
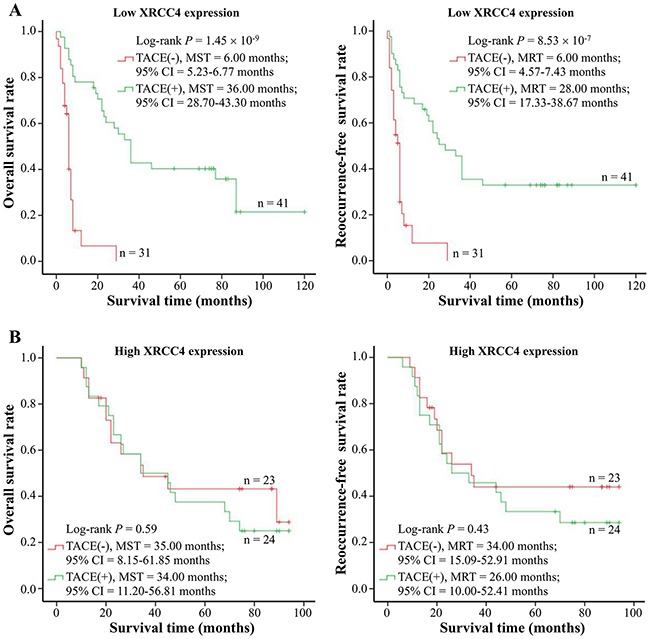
Survival analysis of TACE treatment in strata of XRCC4 expression **(A)** TACE treatment affected overall survival (Left) and tumor reoccurrence-free survival (Right) of hepatocarcinoma cases with low XRCC4 expression (n = 72). **(B)** TACE treatment did not modify overall survival (Left) and tumor reoccurrence-free survival (Right) of hepatocarcinoma cases with high XRCC4 expression. Cumulative hazard function was plotted by Kaplan-Meier's methodology, and *P* value was calculated with two-sided log-rank tests. *Abbreviations*. HXRE, high XRCC4 expression; LXRE, low XRCC4 expression; MST, the median overall survival time; MRT, the median tumor reoccurrence-free survival time.

On the basis of our findings that XRCC4 expression is negatively correlated with microvessel density in the cancerous tissues, we hypothesized that XRCC4 expression could modify the response of cases with hepatocarcinoma to TACE treatment. To address this hypothesis, these patients with hepatocarcinoma in the BCLC-B stage (n = 119) were selected to elucidate the association between XRCC4 expression and the therapeutic value of TACE treatment for hepatocarcinoma (Table 4, Figure 2, and Figure 3). In this analysis, these selected cases were divided into two groups: TACE group who underwent partial resection plus TACE as post-operative adjuvant therapy (n = 65), and non-TACE control group who only accepted tumor surgical resection without post-operative adjuvant TACE treatment (n = 54), according to these patients receiving different initial treatment. As shown in Table 4, there was not significant difference between two groups in terms of the distribution of demographic features (including age, gender, minority, smoking and drinking status, HBV and HCV infective status, and AFP) and pathological characteristics (including liver cirrhosis, tumor size, tumor grade and stage, microvessel density). This suggested the data from two groups were comparable. However, results from survival analyses displayed that TACE treatment as well as XRCC4 expression substantially affected the prognosis of patients with hepatocarcinoma (*P* < 0.05) (Figure 2A and 2B).

**Table 4:** The clinic-pathological features of hepatocarcinoma cases with or without TACE treatment

Variables	Cases, n (%)	TACE treatment, n (%)		χ^2^	*P*
		No	Yes		
Total	119 (100.0)	54 (100.0)	65 (100.0)		
Age (yrs)				0.67	0.41
≤ 48	60 (50.4)	25 (46.3)	35 (53.8)		
> 48	59 (49.6)	29 (53.7)	30 (46.2)		
Sex				0.17	0.68
Man	77 (64.7)	36 (66.7)	41 (63.1)		
Female	42 (35.3)	18 (33.3)	24 (36.9)		
Ethnicity				0.18	0.67
Han	68 (57.1)	32 (59.3)	36 (55.4)		
Zhuang	51 (42.9)	22 (40.7)	29 (44.6)		
HbsAg				0.66	0.42
Negative	31 (26.1)	16 (29.6)	15 (23.1)		
Positive	88 (73.9)	38 (70.4)	50 (76.9)		
anti-HCV				-	1.00^a^
Negative	109 (91.6)	49 (90.7)	60 (92.3)		
Positive	10 (9.4)	5 (9.3)	5 (7.7)		
Smoking status				0.48	0.49
No	81 (68.1)	35 (64.8)	46 (70.8)		
Yes	38 (31.9)	19 (35.2)	19 (29.2)		
Drinking status				0.33	0.57
No	76 (63.9)	33 (61.1)	43 (66.1)		
Yes	43 (36.1)	21 (38.9)	22 (33.9)		
AFP (ng/mL)				0.25	0.62
≤ 20	47 (39.5)	20 (37.0)	27 (41.5)		
> 20	72 (60.5)	34 (63.0)	38 (58.5)		
Liver cirrhosis				0.00	0.97
No	20 (16.8)	9 (16.7)	11 (16.9)		
Yes	99 (83.2)	45 (83.3)	54 (83.1)		
MVD				2.78	0.10
Negative	54 (45.4)	20 (37.0)	34 (52.3)		
Positive	65 (54.6)	34 (63.0)	31 (47.7)		
Tumor grade				0.15	0.70
Low	65 (54.6)	31 (57.4)	35 (53.8)		
High	53 (55.4)	23 (42.6)	30 (46.2)		
XRCC4 expression			0.40	0.53	
Low	72 (60.5)	31 (57.4)	41 (63.1)		
High	47 (39.5)	23 (42.6)	24 (36.9)		

^a^ Fisher's exact test.

More interestingly, the stratified analyses on the basis of different XRCC4 protein levels exhibited that these hepatocarcinoma patients, if with a decreasing XRCC4 expression in their cancerous tissues, would have a relatively better OS and RFS (Figure 3A). However, TACE treatment did not improve the survival of hepatocarcinoma patients with HXRE in their tumor tissues (Figure 3B). Collectively, these data were indicative of the different XRCC4 expression in cancerous tissues differentially modifying therapeutic role of TACE intervention in cases with hepatocarcinoma.

### XRCC4 expression significantly modified the sensitivity of hepatocarcinoma to doxorubicin

**Figure 4:**
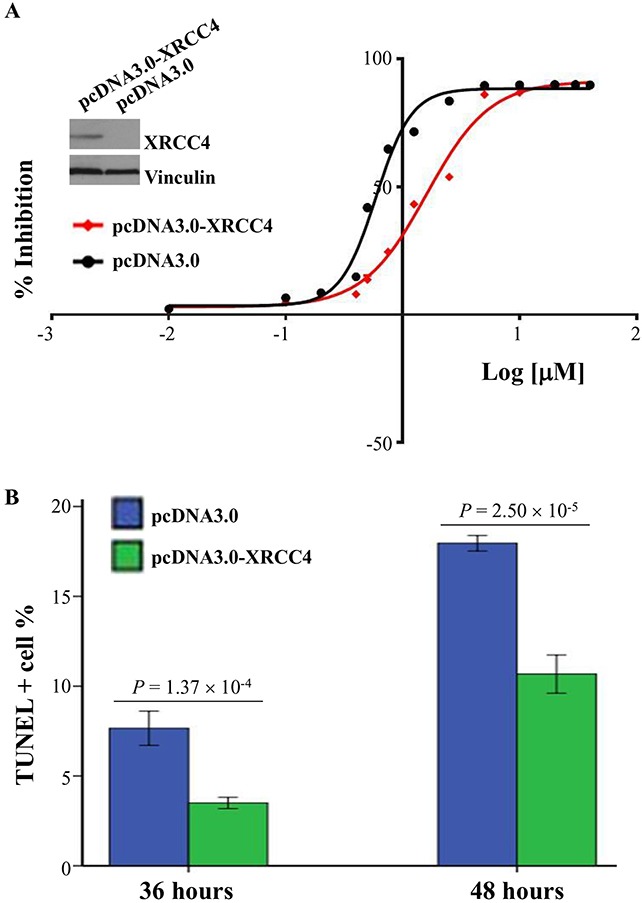
XRCC4 overexpression decreasing the sensitivity of hepatocarcinoma cells SMMC-7721 to doxorubicin treatment *in vitro* SMMC-7721 cells were transfected with pcDNA3.0-XRCC4 or its control pcDNA3.0, and followed by the treatment of doxorubicin. **(A)** The sensitivity of cells to doxorubicin was evaluated by the half-maximal inhibitory concentration (IC50). **(B)** TUNEL staining was used to analyze the doxorubicin-induced cell deaths after 36 and 48 hours of doxorubicin treatment. Data were shown as means ± S.D. and analyzed using *t* test.

**Figure 5:**
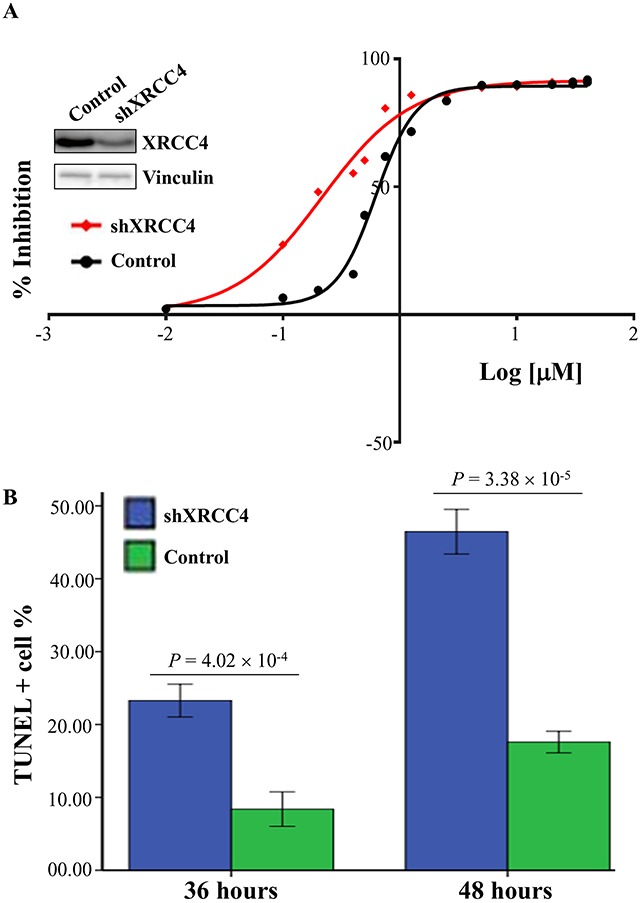
XRCC4 knockdown increasing the sensitivity of hepatocarcinoma cells SMMC-7721 to doxorubicin treatment *in vitro* SMMC-7721 cells were transfected with shRNAs specifically against XRCC4 (shXRCC4, cat#sc-37405) or its control, and followed by the treatment of doxorubicin. **(A)** The sensitivity of cells to doxorubicin was evaluated by the half-maximal inhibitory concentration (IC50). **(B)** TUNEL staining was used to analyze the doxorubicin-induced cell deaths after 36 and 48 hours of doxorubicin treatment. Data were shown as means ± S.D. and analyzed using *t* test.

On the basis of the aforementioned findings that the therapeutic effects of TACE therapy were different among hepatocarcinoma patients with different XRCC4 expression in their tumor tissues, we questioned whether the XRCC4 expression was associated with the sensitivity of hepatocarcinoma cells to anti-cancer drugs such as doxorubicin used in TACE procedure [23]. To approve this, we accomplished a drug sensitivity analysis *in vitro* using anti-cancer drug doxorubicin and hepatocarcinoma cells SMMC-7721. In this analysis, cancer cells were first transfected with the vector expressing XRCC4 or its null vector, and next treated using a series of concentrations of anti-drug (0.01 to 40 μM). Results showed that the half-maximal inhibitory concentration (IC50) and its 95% CIs values of this drug were 1.60 (1.44-1.79) vs. 0.59 (0.55-0.64) μM for these cells with the overexpression of XRCC4 vs. those without overexpression (Figure 4A). TUNEL assay further proved that the overexpression of XRCC4 significantly decreased the death of cancer cells (Figure 4B). Inversely, cancer cells, when XRCC4 expression was knocked down, would feature increasing sensitivity to doxorubicin compared with these without XRCC4 knockdown [IC50s (95% CI), 0.22 (0.18-0.26) vs. 0.62 (0.58-0.67) μM] (Figure 5A and 5B).

### XRCC4 expression correlated with the mutation at codon 247 of XRCC4 gene

Considering that recent two studies have shown that the genic mutation at codon 247 (Ala to Ser, rs3734091) of XRCC4 affects the protein location [24] and hepatocarcinoma prognosis [20], we investigated whether this mutation correlated with XRCC4 expression (Table 5). Higher expression of XRCC4 was detected in the cancerous tissues from hepatocarcinoma patients with wild type of XRCC4 codon 247. Spearman *r* test further exhibited XRCC4 protein expression was negatively related to the mutant type of this gene (*r* = −0.378 and *P* = 9.75 × 10^−16^).

**Table 5:** The association between XRCC4 expression in hepatocarcinoma tissues and the genic mutation at codon 247 of XRCC4

XRCC4 codon 247 Genotype	Low XRCC4 expression (n=180)		High XRCC4 expression (n=241)	
	n	%	n	%
AA	103	57.2	215	89.2
AS	43	23.9	21	8.7
SS	34	18.9	5	2.1

Spearman *r* test: *r* = −0.378 and *P* = 9.75 × 10^−16^. Abbreviation. AA, the homozygote of XRCC4 codon 247 Ala allele; AS, the heterozygote of XRCC4 codon 247 Ala and Ser allele; SS, the homozygote of XRCC4 codon 247 Ser allele.

## DISCUSSION

In this study, we analyzed the correlation between XRCC4 expression and hepatocarcinoma prognosis, and discovered that the downregulation of XRCC4 expression was substantially related to the poor survival of hepatocarcinoma [HRs (95% CIs) 1.63 (1.25-2.11) for OS and 1.55 (1.19-2.02) for RFS, respectively]. Interestingly, decreasing XRCC4 expression in the cancerous tissues improved hepatocarcinoma cases’ therapeutic response to TACE treatment, but increasing expression of XRCC4 did not. These results indicate XRCC4 expression as a valuable marker for hepatocarcinoma survival.

XRCC4, a crucial gene involving in non-homologous end-joining repair and the completion of V(D)J recombination, locates at 5q14.2 and consist of 13 exons. The protein encoded by this gene plays an essential role in DNA double-strand break repair pathway through its scaffold function coupling Ku70/Ku80 heterodimer to DNA ligase IV [16, 25–27]. A decreased ability of repairing damaged DNA due to the dysregulation of XRCC4 may result in genomic instability, genic mutation, and tumor formation [16–19, 28–32]. Recent several studies have shown that the genetic variants of XRCC4 involve in the process of hepatocarcinoma carcinogenesis and affects tumor survival [33, 34]. Our previous reports further displayed that the variants in the C terminal domain of XRCC4 protein were significantly related to progression characteristics of hepatocarcinoma such as tumor size, stage, grade, and differentiation [20, 21, 35]. On the basis of the abovementioned early findings, we collected 421 tissues samples with pathologically diagnosed hepatocarcinoma in Guangxi Area of the People's Republic of China, a known high incidence area of hepatocarcinoma in the world [1]. Results from univariate and multivariate analyses on the basis of Cox Regression Model showed that these hepatocarcinoma cases with different levels of XRCC4 expression in their cancerous tissues would feature different prognosis. Decreasing expression of XRCC4, independent of the clinicopathological features of hepatocarcinoma, increased 1.55-times tumor-recurrence risk and 1.63-folds mortality risk. Collectively, these results implied that downregulation of XRCC4 in the cancerous tissues may be a potential marker for poor prognosis of hepatocarcinoma.

Although it was unclear how reduced level of XRCC4 expression lead to poorer prognosis, our results exhibited this down-regulation of XRCC4 was affected by the structure change resulting from genetic variant at codon 247 of XRCC4, and significantly correlated with increasing micro-vessel density and higher tumor stage. This suggests that XRCC4 expression (including decreasing protein caused by structure change) might involve in tumor progression and angiogenesis. Supporting our findings, recent several studies have displayed that structure change and followed expression change impairs the DNA damage response via dysregulated nuclear localization and can promote carcinogenesis of some tumors such as hepatocarcinoma and breast cancer [20, 21, 24].

Some evidence of XRCC4 expression affecting microvessel density in cancerous tissues and microvessel amounts substantially modifying the therapeutic effects of TACE treatment on hepatocarcinoma [11, 36–38] prompted us to investigate correlation between XRCC4 expression and TACE treatment. The analysis in the data stratified by XRCC4 expression showed that TACE therapy can ameliorate the survival for these hepatocarcinoma patients having LXRE, but not for those with HXRE. Based on these findings, we hypothesized that XRCC4 could change the sensitivity of hepatocarcinoma cells to chemotherapeutic drugs used in TACE procedure. The results from *in vitro* analyses proved this hypothesis. Thus, XRCC4 should be a potential marker for patients with hepatocarcinoma whether to receive TACE treatment or not.

To conclude, this is the first study to describe XRCC4 expression in hepatocarcinoma tissues and its correlation with hepatocarcinoma outcome. We identified XRCC4 could serve as an independent predictor for the clinic outcome in patients with hepatocarcinoma. Particularly, XRCC4 can predict therapeutic value of TACE treatment through modifying the tumor cells’ sensitivity to anti-cancer drugs used in this treatment procedure. Based on these findings, testing XRCC4 expression in the cancerous tissues may help us to form therapeutic strategy for hepatocarcinoma cases. However, the power to elucidate association between XRCC4 expression and TACE therapy was restricted by relatively small sample size. Another important limitation was that we did not do detailed functional analyses for XRCC4 how to modify carcinogenesis and angiogenesis of hepatocarcinoma except for *in vitro* sensitivity assays. Additionally, we did not analyze the expression information of XRCC4 in the non-cancerous tissues. Thus, the molecular mechanism analyses in combination with prospective studies should improve elucidation on the basis of large samples.

## MATERIALS AND METHODS

### Hepatocarcinoma patients

The study protocol was approved by the Institutional Ethics Committee of Youjiang Medical University for Nationalities, and was carried out in accordance with the approved guidelines. This study is a hospital-based retrospective study from Guangxi area, and the design of the Guangxi hepatocarcinoma study has been previously described [11–13]. Briefly, cases were recruited in the affiliated hospitals of Youjiang Medical University for Nationalities and Guangxi Medical University from January 2006 to December 2010. The inclusion criteria on cases are as follows: (1) cases with histopathology-confirmed hepatocarcinoma; (2) cases receiving resect treatment (curative or partial resection) or resect treatment plus post-operative adjuvant TACE as initial treatment according to Chinese Manage Criteria of hepatocarcinoma [39], but not treatment with radiotherapy or chemotherapy before surgical operative treatment; (3) cases understanding the objective of the study and providing informed consent; (4) the ability to complete the necessary investigations and questionnaires; and (5) 5-year follow-up completed and with available cancerous tissue specimens and clinical data. The exclusion criteria consisted of: (1) cases with hepatocarcinoma but not confirmed by histopathological examination; (2) cases receiving chemotherapy or radiotherapy treatment before surgical operative treatment; and (3) cases rejected, dropped out, or lost information.

According to aforementioned inclusion and exclusion criteria, a total of 421 hepatocarcinoma cases, including 228 patients previously studied [20, 21], were included for the present study. Totally, the response rate for the cases has been about 96.5%. After written consent was obtained, the characteristic information of patients, including sex, age, ethnicity, HBV and HCV infection, cirrhosis, tumor size, tumor grade and stage, and treatment information were ascertained as described previously. At the same time, surgically removed tumor samples of all patients with hepatocarcinoma were collected for analyzing XRCC4 expression levels. In this study, those anti-HCV positive and hepatitis B surface antigen (HBsAg) positive in their peripheral serum were defined as groups infected with HCV and HBV. Liver cirrhosis was evaluated by pathological examination. Tumor grade and stage were defined according to Edmondson and Steiner (ES) grading system [40] and the Barcelona Clinic Liver Cancer (BCLC) staging system [4], respectively.

### TACE information

In this retrospective study, post-operative TACE treatment was performed as an important part of the initial treatment for eligible patients with hepatocarcinoma as previously described [11]. Briefly, the inclusion criteria for TACE analysis included: (1) cases with a pathologically diagnosed and BCLC B-stage hepatocarcinoma according to the criteria of “Management of Hepatocellular Carcinoma” [4]; (2) cases having good liver function (Child-Pugh stage A); (3) cases with multiple tumors more than 5 cm or tumor involving a first or second branch of the portal or hepatic veins; (4) the tumor with multiple lesions localized in one lobe of liver, or the main tumor localized in one lobe only with a small solitary lesion in contralateral lobe, or tumor involving a first or second branch of the portal or hepatic vein, which could be safely resected without grossly remaining tumors, and the patient was judged to have well preserved liver function to survive the operation; (5) cases underwent partial hepatectomy, and agreed to post-operative adjuvant TACE treatment; and (6) no contraindication for TACE [11]. The exclusion criteria were as follows: (1) patients with non-hepatocarcinoma on postoperative histopathological examination, serious concurrent medical illness, intractable ascites, tumor recurrence within 4 works after the operation; (2) women cases who were pregnant or breastfeeding; (3) cases rejected, dropped out, or lost information; (4) cases with contraindication for TACE; and (5) cases with history of chemotherapy or radiotherapy treatment before surgical operative treatment [11]. In this study, TACE consisted of an injection containing a mixture of chemotherapeutic agents (doxorubicin at the average dose of 65 mg/m^2^ and cisplatin at the average dose of 7 mg/m^2^) and lipiodol followed by embolization with gelatin foam or polyvinyl alcohol until complete stasis was achieved in the tumor-feeding vessels [11].

### Survival follow-up

For survival analysis, all hepatocarcinoma cases were followed up as described in our previous studies [11–13]. The last follow-up day was set on December 31, 2015, and survival status was confirmed by means of patient or family contact and clinic records. The duration of OS was defined as from the date of the initial treatment to the date of death or last known date alive; whereas RFS was defined as from the date of the initial treatment to the date of tumor recurrence or last known date alive.

### XRCC4 expression assay

The levels of XRCC4 protein in cancerous tissues were analyzed by immunohistochemistry in tissue slides, as previously described [20, 21]. The corresponding anti-XRCC4 polyclonal antibody (dilution 1:500, catalog#sc-8285) and HRP-conjugated secondary antibody (catalog#KIT-9705) were obtained from Santa Cruz Biotechnology, Inc. (Santa Cruz, CA, USA) and Maixin Biotechnology, Inc., respectively. In this study, XRCC4 protein-expressing levels were divided into two classifications: low (the immunoreactive score (IRS), ≤ 4) and high (IRS > 4), according to the median value of IRS systems.

### The mutation at codon 247 of XRCC4 analysis

Genomic DNAs were extracted from tumor tissues of hepatocarcinoma and used to test the genic mutation at codon 247 of XRCC4 by previously published TaqMan-PCR technique [20].

### The micro-vessel density (MVD) evaluation

In the present study, the angiogenesis of cancerous tissues was assessed by the MVD as previously ascribed [41]. Briefly, vessels were stained by CD31 (cat#2011101101, Gene Tech (Shanghai) Company Limited, Shanghai, China) and counted in the cancerous regions over five fields (at × 200 magnification) in each slide. The MVD was defined as positive when the average value of the three readings > 50 [41].

### Cells and culture conditions

The SMMC-7721 cells (a kind of hepatocarcinoma cell line) were purchased from Cell Resource Center of Shanghai Institute for Biological Sciences, Shanghai, China. Cells were cultured in Dulbecco's Modified Eagles Medium (HyClone, Thermo Fisher Scientific (China) CO., Ltd, Shanghai) containing 10% fetal bovine serum (Gibco-Invitrogen Corp., Carlsbad, CA, USA) in atmosphere of 5% CO_2_ at 37 °C using standard techniques. All experimental analyses were done with cells in logarithmic growth. Cells were determined to be free of Mycoplasma [11].

### Plasmid constructs

The full-length XRCC4 expression vector was constructed as previously ascribed [20, 21]. Briefly, the longer fragment (containing full-length XRCC4 cDNA and having terminal *NcoI* and *XhoI* linkers) was first amplified and ligated into the pcDNA3.0 expression vector (Invitrogen, Grand Island, NY, USA). *Escherichia coli* Top 10 cells (Beijing Tiangen Biotech., Co., Ltd., China) were transformed with T4 DNA Ligase (TaKaRa), and colonies were screened for presence of the insert fragment. The resulting plasmid was confirmed by sequencing analysis, and designated as pcDNA3.0-XRCC4_pri_. Similarly, pcDNA3.0 expressing full-length XRCC4 (named as pcDNA3.0-XRCC4) was next constructed using pcDNA3.0-XRCC4_pri_ as the template and GGTAC CATGG AGAGA AAAAT AAGC and CTCGA GTTAA ATCTC ATCAA AGAG as primers. The pcDNA3.0-XRCC4 expression vectors were ascertained by sequencing and western blot.

### Cell sensitivity assay

The sensitivity of SMMC-7721 cells to doxorubicin was elucidated by the half-maximal inhibitory concentration (IC50) using a cell counting kit (CCK-8) assay (cat# CK04, DojindoCorp., Japan) according to our previously published methods [11]. Briefly, a total of 5000 cells were seeded each well in a 96-well plate and transfected with pcDNA3.0-XRCC4 or shRNAs specifically against XRCC4 (shXRCC4, cat#sc-37405) using Lipofectamine™ 2000 transfection reagent (Life Technologies Corporation) following the manufacturer's instructions. The cells were next treated with doxorubicin at 15 different concentrations (0.01-40 μM) (48 hours after transfection). After 36 h of treatment, the CCK-8 solution was added to the well and incubated for 2 hours at 37 °C. Then, the absorbance of optical density (at 450 nm) was recorded, and IC50 values were calculated by nonlinear regression analysis using the GraphPad Prism software with Version 6.0 (GraphPad Software, Inc., San Diego, CA, USA).

### TUNEL assay

Cells were seeded in six-well plates for 24 hours, and then transfected with pcDNA3.0-XRCC4 or shXRCC4 using Lipofectamine™ 2000 transfection reagent (Life Technologies Corporation) following the manufacturer's instructions. After 48 hours, cells were treated with doxorubicin at final concentrations of 1.25 μM for 36 or 48 hours. After that, the cells were all harvested and analyzed by TUNEL staining using an *in situ* cell death detection kit (Roche, Mannheim, Germany) in combination with 4,6-diamino-2-phenyl indole staining. TUNEL-positive cells were counted in at least 300 cells in randomly chosen fields. The data were expressed as a percentage of TUNEL positive cells to total cells [11].

### Statistical analysis

The Pearson χ2 test or Fisher's exact test was used to test differences between groups in the distribution of gender, age, ethnicity, smoking and drinking status, HBV and HCV status, AFP, tumor size, tumor stages and grades, and MVD. Non-conditional logistic regression was used to evaluate odds ratios (ORs) and 95% confidence intervals (CIs) for the effects of XRCC4 expression on the pathological features of hepatocarcinoma. Kaplan–Meier survival analysis (with the log-rank test) was used to evaluate the association between XRCC4 expression and hepatocarcinoma prognosis. Hazard ratios (HRs) and 95% CIs for XRCC4 expression were calculated from univariate and multivariate Cox regression model. In this study, a *P*-value of < 0.05 was considered statistically significant. All analyses were performed with the statistical package for social science (SPSS) version 18 (SPSS Institute, Chicago, IL, USA).

## Abbreviations

XRCC4: X-ray repair complementing 4
TACE: transarterial chemoembolization
ES: Edmondson and Steiner
BCLC: the Barcelona Clinic Liver Cancer
OS: overall survival
RFS: recurrence-free survival
IC50: the half-maximal inhibitory concentration
LXRE: low XRCC4 expression
HXRE: high XRCC4 expression.

## References

[1] Chen W, Zheng R, Baade PD, Zhang S, Zeng H, Bray F, Jemal A, Yu XQ, He J (2016). Cancer statistics in China, 2015. CA Cancer J Clin.

[2] Torre LA, Bray F, Siegel RL, Ferlay J, Lortet-Tieulent J, Jemal A (2015). Global cancer statistics, 2012. CA Cancer J Clin.

[3] Kassahun WT (2016). Contemporary management of fibrolamellar hepatocellular carcinoma: diagnosis, treatment, outcome, prognostic factors, and recent developments. World J Surg Oncol.

[4] Bruix J, Sherman M, American Association for the Study of Liver Diseases (2011). Management of hepatocellular carcinoma: an update. Hepatology.

[5] Ma X, Li RS, Wang J, Huang YQ, Li PY, Wang J, Su HB, Wang RL, Zhang YM, Liu HH, Zhang CE, Ma ZJ, Wang JB (2016). The Therapeutic Efficacy and Safety of Compound Kushen Injection Combined with Transarterial Chemoembolization in Unresectable Hepatocellular Carcinoma: An Update Systematic Review and Meta-Analysis. Front Pharmacol.

[6] Dhir M, Melin AA, Douaiher J, Lin C, Zhen WK, Hussain SM, Geschwind JF, Doyle MB, Abou-Alfa GK, Are C (2016). A Review and Update of Treatment Options and Controversies in the Management of Hepatocellular Carcinoma. Ann Surg.

[7] Sacco R, Mismas V, Marceglia S, Romano A, Giacomelli L, Bertini M, Federici G, Metrangolo S, Parisi G, Tumino E, Bresci G, Corti A, Tredici M (2015). Transarterial radioembolization for hepatocellular carcinoma: An update and perspectives. World J Gastroenterol.

[8] Vilarinho S, Calvisi DF (2014). New advances in precision medicine for hepatocellular carcinoma recurrence prediction and treatment. Hepatology.

[9] Ahn SM, Jang SJ, Shim JH, Kim D, Hong SM, Sung CO, Baek D, Haq F, Ansari AA, Lee SY, Chun SM, Choi S, Choi HJ (2014). Genomic portrait of resectable hepatocellular carcinomas: implications of RB1 and FGF19 aberrations for patient stratification. Hepatology.

[10] Divella R, Daniele A, Abbate I, Savino E, Casamassima P, Sciortino G, Simone G, Gadaleta-Caldarola G, Fazio V, Gadaleta CD, Sabba C, Mazzocca A (2015). Circulating Levels of PAI-1 and SERPINE1 4G/4G Polymorphism Are Predictive of Poor Prognosis in HCC Patients Undergoing TACE. Transl Oncol.

[11] Lu YL, Yao JG, Huang XY, Wang C, Wu XM, Xia Q, Long XD (2016). Prognostic significance of miR-1268a expression and its beneficial effects for post-operative adjuvant transarterial chemoembolization in hepatocellular carcinoma. Sci Rep.

[12] Long XD, Huang XY, Yao JG, Liao P, Tang YJ, Ma Y, Xia Q (2016). Polymorphisms in the precursor microRNAs and aflatoxin B1-related hepatocellular carcinoma. Mol Carcinog.

[13] Huang XY, Yao JG, Huang BC, Ma Y, Xia Q, Long XD (2016). Polymorphisms of a Disintegrin and Metalloproteinase with Thrombospondin Motifs 5 and Aflatoxin B1-Related Hepatocellular Carcinoma. Cancer Epidemiol Biomarkers Prev.

[14] Wu PY, Frit P, Meesala S, Dauvillier S, Modesti M, Andres SN, Huang Y, Sekiguchi J, Calsou P, Salles B, Junop MS (2009). Structural and functional interaction between the human DNA repair proteins DNA ligase IV and XRCC4. Mol Cell Biol.

[15] Pastwa E, Blasiak J (2003). Non-homologous DNA end joining. Acta Biochim Pol.

[16] Wu CN, Liang SY, Tsai CW, Bau DT (2008). The role of XRCC4 in carcinogenesis and anticancer drug discovery. Recent Patents Anticancer Drug Discov.

[17] Takada Y, Someya M, Matsumoto Y, Satoh M, Nakata K, Hori M, Saito M, Hirokawa N, Tateoka K, Teramoto M, Saito T, Hasegawa T, Sakata KI (2016). Influence of Ku86 and XRCC4 expression in uterine cervical cancer on the response to preoperative radiotherapy. Med Mol Morphol.

[18] Hori M, Someya M, Matsumoto Y, Nakata K, Kitagawa M, Hasegawa T, Tsuchiya T, Fukushima Y, Gocho T, Sato Y, Ohnuma H, Kato J, Sugita S (2016). Influence of XRCC4 expression in esophageal cancer cells on the response to radiotherapy. Med Mol Morphol.

[19] Reinardy HC, Bodnar AG (2015). Profiling DNA damage and repair capacity in sea urchin larvae and coelomocytes exposed to genotoxicants. Mutagenesis.

[20] Long XD, Zhao D, Wang C, Huang XY, Yao JG, Ma Y, Wei ZH, Liu M, Zeng LX, Mo XQ, Zhang JJ, Xue F, Zhai B (2013). Genetic Polymorphisms in DNA Repair Genes XRCC4 and XRCC5 and Aflatoxin B1-related Hepatocellular Carcinoma. Epidemiology.

[21] Long XD, Yao JG, Zeng Z, Ma Y, Huang XY, Wei ZH, Liu M, Zhang JJ, Xue F, Zhai B, Xia Q (2013). Polymorphisms in the coding region of X-ray repair complementing group 4 and aflatoxin B1-related hepatocellular carcinoma. Hepatology.

[22] Wu XM, Xi ZF, Liao P, Huang HD, Huang XY, Wang C, Ma Y, Xia Q, Yao JG, Long XD (2017). Diagnostic and prognostic potential of serum microRNA-4651 for patients with hepatocellular carcinoma related to aflatoxin B1. Oncotarget.

[23] Vogl TJ, Naguib NN, Nour-Eldin NE, Rao P, Emami AH, Zangos S, Nabil M, Abdelkader A (2009). Review on transarterial chemoembolization in hepatocellular carcinoma: palliative, combined, neoadjuvant, bridging, and symptomatic indications. Eur J Radiol.

[24] He M, Hu X, Chen L, Cao AY, Yu KD, Shi TY, Kuang XY, Shi WB, Ling H, Li S, Qiao F, Yao L, Wei Q (2014). A recessive variant of XRCC4 predisposes to non- BRCA1/2 breast cancer in chinese women and impairs the DNA damage response via dysregulated nuclear localization. Oncotarget.

[25] Brouwer I, Sitters G, Candelli A, Heerema SJ, Heller I, de AJ Melo, Zhang H, Normanno D, Modesti M, Peterman EJ, Wuite GJ (2016). Sliding sleeves of XRCC4-XLF bridge DNA and connect fragments of broken DNA. Nature.

[26] Shao N, Jiang WY, Qiao D, Zhang SG, Wu Y, Zhang XX, Hua LX, Ding Y, Feng NH (2012). An updated meta-analysis of XRCC4 polymorphisms and cancer risk based on 31 case-control studies. Cancer Biomark.

[27] Sibanda BL, Critchlow SE, Begun J, Pei XY, Jackson SP, Blundell TL, Pellegrini L (2001). Crystal structure of an Xrcc4-DNA ligase IV complex. Nat Struct Biol.

[28] Junop MS, Modesti M, Guarne A, Ghirlando R, Gellert M, Yang W (2000). Crystal structure of the Xrcc4 DNA repair protein and implications for end joining. EMBO J.

[29] Li XL, Meng QH, Fan SJ (2009). Adenovirus-mediated expression of UHRF1 reduces the radiosensitivity of cervical cancer HeLa cells to gamma-irradiation. Acta Pharmacol Sin.

[30] Pieraccioli M, Nicolai S, Antonov A, Somers J, Malewicz M, Melino G, Raschella G (2016). ZNF281 contributes to the DNA damage response by controlling the expression of XRCC2 and XRCC4. Oncogene.

[31] Shao N, Li J, Xu B, Wang Y, Lu X, Feng N (2014). Role of the functional variant (−652T>G) in the XRCC4 promoter in prostate cancer. Mol Biol Rep.

[32] Myers MV, Maxwell SE, Wang X (2014). Quantification of XRCC and DNA-PK proteins in cancer cell lines and human tumors by LC-MS/MS. Bioanalysis.

[33] Makkoch J, Praianantathavorn K, Sopipong W, Chuaypen N, Tangkijvanich P, Payungporn S (2016). Genetic Variations in XRCC4 (rs1805377) and ATF6 (rs2070150) are not Associated with Hepatocellular Carcinoma in Thai Patients with Hepatitis B Virus Infection. Asian Pac J Cancer Prev.

[34] Jung SW, Park NH, Shin JW, Park BR, Kim CJ, Lee JE, Shin ES, Kim JA, Chung YH (2012). Polymorphisms of DNA repair genes in Korean hepatocellular carcinoma patients with chronic hepatitis B: possible implications on survival. J Hepatol.

[35] Yao JG, Huang XY, Long XD (2014). Interaction of DNA repair gene polymorphisms and aflatoxin B1 in the risk of hepatocellular carcinoma. Int J Clin Exp Pathol.

[36] Yi J, Liao X, Yang Z, Li X (2001). Study on the changes in microvessel density in hepatocellular carcinoma following transcatheter arterial chemoembolization. J Tongji Med Univ.

[37] Jiao HK, Xu Y, Li J, Wang W, Mei Z, Long XD, Chen GQ (2015). Prognostic significance of Cbx4 expression and its beneficial effect for transarterial chemoembolization in hepatocellular carcinoma. Cell Death Dis.

[38] Li J, Xu Y, Long XD, Wang W, Jiao HK, Mei Z, Yin QQ, Ma LN, Zhou AW, Wang LS, Yao M, Xia Q, Chen GQ (2014). Cbx4 governs HIF-1alpha to potentiate angiogenesis of hepatocellular carcinoma by its SUMO E3 ligase activity. Cancer Cell.

[39] CSLC, CSCO (2009). Diagnosis and treatment of primary liver cancer: a standardized expert consensus. Chinese Clinical Oncology.

[40] Dabbs DJ, Geisinger KR, Ruggiero F, Raab SS, Nalesnik M, Silverman JF, Association of Directors of Anatomic and Surgical Pathology (2004). Recommendations for the reporting of tissues removed as part of the surgical treatment of malignant liver tumors. Hum Pathol.

[41] Liu YX, Long XD, Xi ZF, Ma Y, Huang XY, Yao JG, Wang C, Xing TY, Xia Q (2014). MicroRNA-24 modulates aflatoxin B1-related hepatocellular carcinoma prognosis and tumorigenesis. Biomed Res Int.

